# 1-{2-[(2-hydroxybenzylidene)-amino]-ethyl}-3-methyl-3*H*-imidazolium hexafluorophosphate

**DOI:** 10.1107/S1600536808037124

**Published:** 2008-11-13

**Authors:** Bin Li, Yi-Qun Li, Yue-Peng Cai, Mei-Yun Zhou

**Affiliations:** a Department of Chemistry, Jinan University, Guangzhou, Guangdong 510632, People’s Republic of China; b School of Chemistry and the Environment, South China Normal University, Guangzhou, Guangdong 510631, People’s Republic of China

## Abstract

The title Schiff base compound, C_13_H_16_N_3_O^+^·PF_6_^−^, was derived from the condensation of 2-hydroxy­benaldehyde with the ionic liquid 1-(2-amino­ethyl)-3-methyl­imidazolium hexa­fluoro­phosphate in an ethanol solution. The asymmetric unit comprises one cation and two PF_6_^−^ anions. The dihedral angle between the aromatic and imidazole rings is 15.2 (2)°. An intra­molecular O—H⋯N hydrogen bond is found which generates an *S*(6) ring motif.

## Related literature

For the synthesis of Schiff bases, see: Pradeep (2005 ▶); Butcher *et al.* (2005 ▶). For background on ionic liquids and their applications, see: Cai *et al.* (2006 ▶); Peng & Song (2006 ▶).
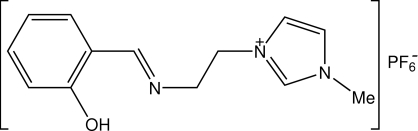


## Experimental

### 

#### Crystal data

C_13_H_16_N_3_O^+^·PF_6_^−^
*M* *_r_* = 375.26
Monoclinic, 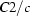

*a* = 28.239 (15) Å
*b* = 7.134 (4) Å
*c* = 18.017 (9) Å
β = 118.342 (6)°
*V* = 3194 (3) Å^3^
*Z* = 8
Mo *K*α radiation
μ = 0.24 mm^−1^
*T* = 298 (2) K
0.32 × 0.25 × 0.15 mm

#### Data collection

Bruker SMART CCD area-detector diffractometer
Absorption correction: multi-scan (*SADABS*; Sheldrick, 1996 ▶) *T*_min_ = 0.926, *T*_max_ = 0.965
8091 measured reflections
2969 independent reflections
1965 reflections with *I* > 2σ(*I*)
*R*_int_ = 0.043

#### Refinement

*R*[*F* ^2^ > 2σ(*F* ^2^)] = 0.066
*wR*(*F* ^2^) = 0.215
*S* = 1.01
2969 reflections
221 parameters
H-atom parameters constrained
Δρ_max_ = 0.72 e Å^−3^
Δρ_min_ = −0.29 e Å^−3^

### 

Data collection: *SMART* (Bruker, 1998 ▶); cell refinement: *SAINT* (Bruker, 1999 ▶); data reduction: *SAINT*; program(s) used to solve structure: *SHELXS97* (Sheldrick, 2008 ▶); program(s) used to refine structure: *SHELXL97* (Sheldrick, 2008 ▶); molecular graphics: *SHELXTL* (Sheldrick, 2008 ▶); software used to prepare material for publication: *SHELXTL*.

## Supplementary Material

Crystal structure: contains datablocks I, global. DOI: 10.1107/S1600536808037124/tk2322sup1.cif

Structure factors: contains datablocks I. DOI: 10.1107/S1600536808037124/tk2322Isup2.hkl

Additional supplementary materials:  crystallographic information; 3D view; checkCIF report

## Figures and Tables

**Table 1:** Hydrogen-bond geometry (Å, °)

*D*—H⋯*A*	*D*—H	H⋯*A*	*D*⋯*A*	*D*—H⋯*A*
O1—H1⋯N1	0.82	1.85	2.572 (5)	147

**Fig. 1.**
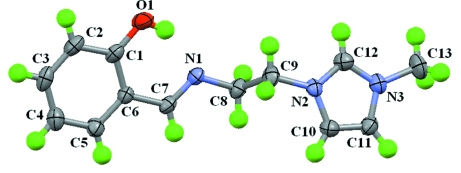
The molecular structure of the cation in (I) showing the atom numbering Scheme. Displacement ellipsoids are drawn at the 50% probability level.

## supplementary crystallographic information

### Comment

The use of functionalized ionic liquids continues to receive attention in chemical synthesis and engineering, including as catalysts in organic synthesis (Cai *et al.*, 2006; Peng & Song, 2006). Schiff base compounds are one of most prevalent mixed-donor ligands in the field of coordination chemistry (Pradeep, 2005; Butcher *et al.*, 2005). Herein, we report the crystal structure of the title salt, (I).

Compound (I) is a Schiff base formed from the reaciton of 2-hydroxybenaldehyde and ionic liquid 1-(2-aminoethyl)-3-methylimidazolium hexafluorophosphate. The molecular structure of the cation is shown in Fig. 1. The aromatic and imidazole rings form a dihedral angle of 15.2 (2)°. In the cation, an intramolecular O1—H1···N1 hydrogen bond leads to a six-membered ring S(6) motif, Table 1.

### Experimental

A mixture of the ionic liquid 1-(2-aminoethyl)-3-methylimidazolium hexafluorophosphate (5 mmol) and 2-hydroxybenzaldehyde (5 mmol) in ethanol was stirred for 4 h. After the completion of the reaction, the excess ethanol was removed by distillation. The colorless solid obtained was filtered and washed with ethanol. Single crystals suitable for X-ray diffraction were obtained by slow evaporation of an ethyl acetate solution of (I) at room temperature.

### Refinement

The H atom bound to O1 was located from a difference Fourier map and refined as riding, with O—H = 0.82 Å, and with *U*_iso_(H) = 1.5 *U*_eq_(O). The remaining H atoms were located in a difference syntheses and refined with C—H = 0.93–0.97 Å, and with *U*_iso_(H) = 1.2 - 1.5*U*_eq_(C)].

### Crystal data

**Table:**

C_13_H_16_N_3_O^+^·PF_6_^–^	*F*_000_ = 1536
*M**_r_* = 375.26	*D*_x_ = 1.561 Mg m^−^^3^
Monoclinic, *C*2/*c*	Mo *K*α radiation λ = 0.71073 Å
Hall symbol: -C 2yc	Cell parameters from 2060 reflections
*a* = 28.239 (15) Å	θ = 2.9–22.9º
*b* = 7.134 (4) Å	µ = 0.24 mm^−^^1^
*c* = 18.017 (9) Å	*T* = 298 (2) K
β = 118.342 (6)º	Prism, yellow
*V* = 3194 (3) Å^3^	0.32 × 0.25 × 0.15 mm
*Z* = 8	

### Data collection

**Table:**

Bruker SMART CCD area-detector diffractometer	2969 independent reflections
Radiation source: fine-focus sealed tube	1965 reflections with *I* > 2σ(*I*)
Monochromator: graphite	*R*_int_ = 0.043
*T* = 298(2) K	θ_max_ = 25.5º
φ and ω scans	θ_min_ = 2.3º
Absorption correction: multi-scan(SADABS; Sheldrick, 1996)	*h* = −19→34
*T*_min_ = 0.926, *T*_max_ = 0.965	*k* = −8→8
8091 measured reflections	*l* = −21→19

### Refinement

**Table:**

Refinement on *F*^2^	Secondary atom site location: difference Fourier map
Least-squares matrix: full	Hydrogen site location: inferred from neighbouring sites
*R*[*F*^2^ > 2σ(*F*^2^)] = 0.066	H-atom parameters constrained
*wR*(*F*^2^) = 0.215	*w* = 1/[σ^2^(*F*_o_^2^) + (0.095*P*)^2^ + 15.5678*P*] where *P* = (*F*_o_^2^ + 2*F*_c_^2^)/3
*S* = 1.01	(Δ/σ)_max_ < 0.001
2969 reflections	Δρ_max_ = 0.72 e Å^−^^3^
221 parameters	Δρ_min_ = −0.29 e Å^−^^3^
Primary atom site location: structure-invariant direct methods	Extinction correction: none

### Special details

**Table:**

Geometry. All e.s.d.'s (except the e.s.d. in the dihedral angle between two l.s. planes) are estimated using the full covariance matrix. The cell e.s.d.'s are taken into account individually in the estimation of e.s.d.'s in distances, angles and torsion angles; correlations between e.s.d.'s in cell parameters are only used when they are defined by crystal symmetry. An approximate (isotropic) treatment of cell e.s.d.'s is used for estimating e.s.d.'s involving l.s. planes.
Refinement. Refinement of *F*^2^ against ALL reflections. The weighted *R*-factor *wR* and goodness of fit *S* are based on *F*^2^, conventional *R*-factors *R* are based on *F*, with *F* set to zero for negative *F*^2^. The threshold expression of *F*^2^ > σ(*F*^2^) is used only for calculating *R*-factors(gt) *etc*. and is not relevant to the choice of reflections for refinement. *R*-factors based on *F*^2^ are statistically about twice as large as those based on *F*, and *R*- factors based on ALL data will be even larger.

### Fractional atomic coordinates and isotropic or equivalent isotropic displacement parameters (Å^2^)

**Table:**

	*x*	*y*	*z*	*U*_iso_*/*U*_eq_	
P1	0.7500	0.7500	0.0000	0.0501 (5)	
P2	1.0000	0.6525 (3)	0.2500	0.0583 (5)	
F1	0.81350 (11)	0.7434 (5)	0.05733 (18)	0.0696 (9)	
F2	0.74441 (13)	0.5737 (5)	0.04985 (19)	0.0742 (9)	
F3	0.74598 (13)	0.8887 (5)	0.06617 (18)	0.0737 (9)	
F4	1.0089 (2)	0.5007 (9)	0.3170 (3)	0.159 (2)	
F5	0.93929 (17)	0.6504 (10)	0.2199 (4)	0.162 (2)	
F6	1.0081 (3)	0.8057 (8)	0.3156 (3)	0.156 (2)	
O1	0.80570 (15)	0.2094 (7)	0.2303 (3)	0.0801 (12)	
H1	0.8137	0.1973	0.1923	0.120*	
N1	0.86812 (17)	0.1728 (6)	0.1638 (3)	0.0551 (10)	
N2	0.86519 (16)	0.3299 (5)	−0.0349 (2)	0.0513 (10)	
N3	0.85173 (18)	0.3101 (6)	−0.1622 (3)	0.0591 (11)	
C1	0.8504 (2)	0.1958 (7)	0.3054 (3)	0.0566 (12)	
C2	0.8461 (3)	0.2063 (8)	0.3792 (4)	0.0671 (15)	
H2	0.8126	0.2218	0.3762	0.081*	
C3	0.8914 (3)	0.1938 (8)	0.4566 (4)	0.0722 (16)	
H3	0.8882	0.2028	0.5055	0.087*	
C4	0.9408 (3)	0.1686 (8)	0.4625 (4)	0.0719 (16)	
H4	0.9710	0.1597	0.5152	0.086*	
C5	0.9460 (2)	0.1563 (7)	0.3911 (3)	0.0626 (13)	
H5	0.9799	0.1381	0.3956	0.075*	
C6	0.90111 (19)	0.1708 (6)	0.3114 (3)	0.0491 (11)	
C7	0.9077 (2)	0.1593 (7)	0.2363 (3)	0.0536 (12)	
H7	0.9419	0.1416	0.2420	0.064*	
C8	0.8777 (2)	0.1599 (7)	0.0908 (3)	0.0600 (13)	
H8A	0.8596	0.0508	0.0571	0.072*	
H8B	0.9160	0.1475	0.1095	0.072*	
C9	0.8567 (2)	0.3333 (8)	0.0397 (3)	0.0634 (14)	
H9A	0.8185	0.3450	0.0217	0.076*	
H9B	0.8748	0.4416	0.0741	0.076*	
C10	0.9137 (2)	0.3503 (8)	−0.0329 (3)	0.0612 (13)	
H10	0.9466	0.3686	0.0150	0.073*	
C11	0.9053 (2)	0.3392 (8)	−0.1120 (3)	0.0627 (13)	
H11	0.9312	0.3494	−0.1297	0.075*	
C12	0.8285 (2)	0.3056 (7)	−0.1133 (3)	0.0612 (13)	
H12	0.7920	0.2881	−0.1319	0.073*	
C13	0.8247 (3)	0.2925 (10)	−0.2541 (3)	0.087 (2)	
H13A	0.7873	0.2649	−0.2742	0.131*	
H13B	0.8410	0.1930	−0.2699	0.131*	
H13C	0.8280	0.4080	−0.2786	0.131*	

### Atomic displacement parameters (Å^2^)

**Table:**

	*U*^11^	*U*^22^	*U*^33^	*U*^12^	*U*^13^	*U*^23^
P1	0.0469 (10)	0.0621 (11)	0.0450 (9)	0.0109 (8)	0.0248 (8)	0.0034 (8)
P2	0.0526 (11)	0.0736 (13)	0.0541 (10)	0.000	0.0296 (9)	0.000
F1	0.0422 (16)	0.088 (2)	0.0708 (19)	0.0081 (15)	0.0202 (14)	−0.0014 (16)
F2	0.084 (2)	0.075 (2)	0.0696 (19)	0.0024 (17)	0.0408 (17)	0.0159 (16)
F3	0.084 (2)	0.082 (2)	0.0611 (18)	0.0150 (17)	0.0393 (17)	−0.0107 (15)
F4	0.181 (5)	0.157 (5)	0.135 (4)	−0.002 (4)	0.073 (4)	0.072 (4)
F5	0.063 (3)	0.243 (7)	0.174 (5)	−0.004 (3)	0.051 (3)	−0.033 (5)
F6	0.209 (6)	0.150 (5)	0.108 (4)	−0.003 (4)	0.076 (4)	−0.052 (3)
O1	0.055 (2)	0.116 (3)	0.080 (3)	−0.006 (2)	0.040 (2)	−0.018 (2)
N1	0.058 (3)	0.061 (2)	0.057 (2)	0.001 (2)	0.035 (2)	−0.002 (2)
N2	0.056 (2)	0.051 (2)	0.046 (2)	0.0114 (18)	0.0238 (19)	0.0029 (17)
N3	0.069 (3)	0.056 (2)	0.051 (2)	0.004 (2)	0.027 (2)	0.0005 (19)
C1	0.062 (3)	0.053 (3)	0.070 (3)	−0.009 (2)	0.043 (3)	−0.004 (2)
C2	0.078 (4)	0.063 (3)	0.089 (4)	−0.009 (3)	0.062 (4)	−0.006 (3)
C3	0.111 (5)	0.056 (3)	0.077 (4)	−0.003 (3)	0.067 (4)	0.005 (3)
C4	0.092 (4)	0.068 (4)	0.060 (3)	0.007 (3)	0.040 (3)	0.009 (3)
C5	0.064 (3)	0.063 (3)	0.063 (3)	0.011 (3)	0.033 (3)	0.010 (3)
C6	0.056 (3)	0.046 (2)	0.053 (3)	0.002 (2)	0.031 (2)	0.003 (2)
C7	0.057 (3)	0.050 (3)	0.069 (3)	0.005 (2)	0.042 (3)	0.005 (2)
C8	0.072 (3)	0.061 (3)	0.062 (3)	0.003 (3)	0.044 (3)	−0.006 (2)
C9	0.079 (4)	0.065 (3)	0.057 (3)	0.016 (3)	0.042 (3)	0.003 (2)
C10	0.049 (3)	0.068 (3)	0.063 (3)	0.003 (2)	0.024 (2)	0.003 (3)
C11	0.065 (3)	0.067 (3)	0.067 (3)	0.006 (3)	0.040 (3)	0.008 (3)
C12	0.056 (3)	0.062 (3)	0.066 (3)	−0.001 (2)	0.029 (3)	−0.005 (3)
C13	0.105 (5)	0.101 (5)	0.047 (3)	0.002 (4)	0.029 (3)	−0.005 (3)

### Geometric parameters (Å, °)

**Table:**

P1—F1^i^	1.589 (3)	C1—C6	1.396 (7)
P1—F1	1.589 (3)	C2—C3	1.377 (8)
P1—F3	1.594 (3)	C2—H2	0.9300
P1—F3^i^	1.594 (3)	C3—C4	1.359 (8)
P1—F2^i^	1.596 (3)	C3—H3	0.9300
P1—F2	1.596 (3)	C4—C5	1.366 (7)
P2—F5^ii^	1.533 (4)	C4—H4	0.9300
P2—F5	1.533 (4)	C5—C6	1.398 (7)
P2—F6	1.544 (5)	C5—H5	0.9300
P2—F6^ii^	1.544 (5)	C6—C7	1.454 (6)
P2—F4^ii^	1.550 (5)	C7—H7	0.9300
P2—F4	1.550 (5)	C8—C9	1.487 (7)
O1—C1	1.346 (6)	C8—H8A	0.9700
O1—H1	0.8200	C8—H8B	0.9700
N1—C7	1.256 (6)	C9—H9A	0.9700
N1—C8	1.467 (6)	C9—H9B	0.9700
N2—C12	1.308 (6)	C10—C11	1.333 (7)
N2—C10	1.360 (6)	C10—H10	0.9300
N2—C9	1.474 (6)	C11—H11	0.9300
N3—C12	1.324 (6)	C12—H12	0.9300
N3—C11	1.360 (7)	C13—H13A	0.9600
N3—C13	1.464 (6)	C13—H13B	0.9600
C1—C2	1.392 (7)	C13—H13C	0.9600
			
F1^i^—P1—F1	180.00 (12)	C1—C2—H2	119.9
F1^i^—P1—F3	90.50 (16)	C4—C3—C2	120.9 (5)
F1—P1—F3	89.50 (16)	C4—C3—H3	119.6
F1^i^—P1—F3^i^	89.50 (16)	C2—C3—H3	119.6
F1—P1—F3^i^	90.50 (16)	C3—C4—C5	120.1 (6)
F3—P1—F3^i^	180.0 (2)	C3—C4—H4	120.0
F1^i^—P1—F2^i^	89.62 (17)	C5—C4—H4	120.0
F1—P1—F2^i^	90.38 (17)	C4—C5—C6	120.8 (5)
F3—P1—F2^i^	89.60 (17)	C4—C5—H5	119.6
F3^i^—P1—F2^i^	90.40 (17)	C6—C5—H5	119.6
F1^i^—P1—F2	90.38 (17)	C1—C6—C5	119.1 (4)
F1—P1—F2	89.62 (17)	C1—C6—C7	121.1 (5)
F3—P1—F2	90.40 (17)	C5—C6—C7	119.8 (4)
F3^i^—P1—F2	89.60 (17)	N1—C7—C6	121.3 (4)
F2^i^—P1—F2	180.0 (2)	N1—C7—H7	119.4
F5^ii^—P2—F5	178.9 (5)	C6—C7—H7	119.4
F5^ii^—P2—F6	90.1 (4)	N1—C8—C9	108.2 (4)
F5—P2—F6	90.7 (3)	N1—C8—H8A	110.1
F5^ii^—P2—F6^ii^	90.7 (3)	C9—C8—H8A	110.1
F5—P2—F6^ii^	90.1 (3)	N1—C8—H8B	110.1
F6—P2—F6^ii^	89.9 (5)	C9—C8—H8B	110.1
F5^ii^—P2—F4^ii^	90.6 (3)	H8A—C8—H8B	108.4
F5—P2—F4^ii^	88.6 (3)	N2—C9—C8	111.1 (4)
F6—P2—F4^ii^	179.0 (4)	N2—C9—H9A	109.4
F6^ii^—P2—F4^ii^	89.3 (3)	C8—C9—H9A	109.4
F5^ii^—P2—F4	88.6 (3)	N2—C9—H9B	109.4
F5—P2—F4	90.6 (3)	C8—C9—H9B	109.4
F6—P2—F4	89.3 (3)	H9A—C9—H9B	108.0
F6^ii^—P2—F4	179.0 (4)	C11—C10—N2	107.4 (5)
F4^ii^—P2—F4	91.4 (5)	C11—C10—H10	126.3
C1—O1—H1	109.5	N2—C10—H10	126.3
C7—N1—C8	118.3 (4)	C10—C11—N3	107.3 (5)
C12—N2—C10	108.3 (4)	C10—C11—H11	126.4
C12—N2—C9	126.8 (5)	N3—C11—H11	126.4
C10—N2—C9	124.9 (4)	N2—C12—N3	109.1 (5)
C12—N3—C11	107.9 (4)	N2—C12—H12	125.5
C12—N3—C13	126.4 (5)	N3—C12—H12	125.5
C11—N3—C13	125.7 (5)	N3—C13—H13A	109.5
O1—C1—C2	119.5 (5)	N3—C13—H13B	109.5
O1—C1—C6	121.6 (4)	H13A—C13—H13B	109.5
C2—C1—C6	118.9 (5)	N3—C13—H13C	109.5
C3—C2—C1	120.3 (5)	H13A—C13—H13C	109.5
C3—C2—H2	119.9	H13B—C13—H13C	109.5
			
O1—C1—C2—C3	−179.6 (5)	C7—N1—C8—C9	−123.0 (5)
C6—C1—C2—C3	0.6 (8)	C12—N2—C9—C8	107.3 (6)
C1—C2—C3—C4	−0.9 (8)	C10—N2—C9—C8	−72.1 (7)
C2—C3—C4—C5	0.4 (9)	N1—C8—C9—N2	179.9 (4)
C3—C4—C5—C6	0.5 (9)	C12—N2—C10—C11	0.5 (6)
O1—C1—C6—C5	−179.6 (5)	C9—N2—C10—C11	180.0 (5)
C2—C1—C6—C5	0.3 (7)	N2—C10—C11—N3	−0.6 (6)
O1—C1—C6—C7	0.3 (7)	C12—N3—C11—C10	0.5 (6)
C2—C1—C6—C7	−179.9 (5)	C13—N3—C11—C10	178.9 (5)
C4—C5—C6—C1	−0.8 (8)	C10—N2—C12—N3	−0.1 (6)
C4—C5—C6—C7	179.3 (5)	C9—N2—C12—N3	−179.6 (4)
C8—N1—C7—C6	−180.0 (4)	C11—N3—C12—N2	−0.2 (6)
C1—C6—C7—N1	0.5 (7)	C13—N3—C12—N2	−178.6 (5)
C5—C6—C7—N1	−179.7 (5)		

Symmetry codes: (i) −*x*+3/2, −*y*+3/2, −*z*; (ii) −*x*+2, *y*, −*z*+1/2.

### Hydrogen-bond geometry (Å, °)

**Table:**

*D*—H···*A*	*D*—H	H···*A*	*D*···*A*	*D*—H···*A*
O1—H1···N1	0.82	1.85	2.572 (5)	147
